# Cross-sector Service Provision in Health and Social Care: An Umbrella Review

**DOI:** 10.5334/ijic.2460

**Published:** 2016-04-08

**Authors:** Shannon Winters, Lilian Magalhaes, Elizabeth Anne Kinsella, Anita Kothari

**Affiliations:** Health and Rehabilitation Sciences, Faculty of Health Sciences, Western University, London, ON, Canada; George and Fay Yee Centre for Healthcare Innovation Evaluation Platform, Winnipeg Regional Health Authority and The University of Manitoba, Winnipeg, MB, Canada; Health and Rehabilitation Sciences, Faculty of Health Sciences, Western University, London, ON, Canada; Health and Rehabilitation Sciences, Faculty of Health Sciences, Western University, London, ON, Canada; Centre for Education Research and Innovation, Schulich School of Medicine & Dentistry, Western University, London, ON, Canada

**Keywords:** cross-sector collaboration, service provision, health care, social care, partnership

## Abstract

**Introduction:**

Meeting the complex health needs of people often requires interaction among numerous different sectors. No one service can adequately respond to the diverse care needs of consumers. Providers working more effectively together is frequently touted as the solution. Cross-sector service provision is defined as independent, yet interconnected sectors working together to better meet the needs of consumers and improve the quality and effectiveness of service provision. Cross-sector service provision is expected, yet much remains unknown about how it is conceptualised or its impact on health status. This umbrella review aims to clarify the critical attributes that shape cross-sector service provision by presenting the current state of the literature and building on the findings of the 2004 review by Sloper.

**Methods:**

Literature related to cross-sector service provision is immense, which poses a challenge for decision makers wishing to make evidence-informed decisions. An umbrella review was conducted to articulate the overall state of cross-sector service provision literature and examine the evidence to allow for the discovery of consistencies and discrepancies across the published knowledge base.

**Findings:**

Sixteen reviews met the inclusion criteria. Seven themes emerged: Focusing on the consumer, developing a shared vision of care, leadership involvement, service provision across the boundaries, adequately resourcing the arrangement, developing novel arrangements or aligning with existing relationships, and strengthening connections between sectors. Future research from a cross-organisational, rather than individual provider, perspective is needed to better understand what shapes cross-sector service provision at the boundaries.

**Conclusion:**

Findings aligned closely with the work done by Sloper and raise red flags related to reinventing what is already known. Future researchers should look to explore novel areas rather than looking into areas that have been explored at length. Evaluations of out-comes related to cross-sector service provision are still needed before any claims about effectiveness can be made.

## Introduction

Meeting the complex health needs of people often requires interaction among numerous different sectors [1,2]. The need for various sectors to work together to offer continuous, coordinated and effective care has been depicted as critical [1,2,3]. If sectors are unable, unwilling or precluded from working together, the consumer may not receive the care they require, potentially resulting in dire consequences [1,2]. It has been repeatedly said in the literature that no one service can adequately respond to the diverse needs of the healthcare consumer [1,4]. Enhancing the ability for providers to work together is frequently touted as the solution to this problem [5]. As Kodner [2] states, performance suffers if integration is absent at various levels; furthermore, services are delayed and quality and patient satisfaction decline [1,2]. As Glasby and Dickinson [1] emphasise, a lack of partnership and co-ordination can literally be a matter of life and death, with fatal outcomes resulting from sectors not working together to meet the complex needs of consumers.

When sectors within the health and social care industries work together to provide a service, it is said to enhance the quality of service provision by providing more consistent, coordinated, appropriate care in a more timely fashion [2]. Additionally, as Kernaghan [5] notes, cross-sector service provision has evolved from impromptu responses to more concerted and planned approaches to increasing efficiency, effectiveness and responsiveness of organisations. Moreover, Koder [2] indicated that cross-sector service provision can facilitate less duplication and waste, more flexible service provision and better coordination and continuity.

The call for cross-sector service provision, as found in many high-level international, national and local policies [1,5] mandates that sectors will work together to provide better care. Numerous assumptions exist within these policies, the most striking being that cross-sector service provision does in fact improve the care that gets delivered [1,6]. Cross-sector service provision is now seen as the expectation rather than the exception [1] and is becoming increasingly more common [5]. However, based on the previous literature in the area, much remains unknown about how cross-sector service provision is conceptualised, let alone its presumed positive impact [6,7]. The push to deliver more strongly coordinated services across sectors claims to be based on evidence; however, these claims may be hollow given that what the existing evidence states is that very little is known about the impact of cross-sector service provision [3,6,8,9]. In fact Babiak and Thibault [6] found that there were an increasing number of studies pointing to challenges, rather than benefits related to cross-sector partnerships and that more work was needed to determine how these challenges could be overcome. Pronouncements to minimise the boundaries between sectors within the healthcare and social care industries have advanced more rapidly than the available evidence supports. Considering the issues mentioned above, more research is warranted to establish the benefits of cross-sector service provision prior to introducing major changes. The current umbrella review aims to clarify the essential attributes that shape cross-sector service provision by critically examining the current state of the literature and building on the findings of the 2004 review of ‘coordinated multi-agency working’ conducted by Sloper [10].

In this article, the authors generated the term cross-sector service provision to refer to independent, yet interconnected sectors^1^ working together to better meet the needs of consumers and improve the quality and effectiveness of service provision. We will consistently use cross-sector service provision with the understanding that numerous substantial and independent bodies of research inform the concept. Our focus is on what many refer to as integration, collaboration, partnership and coordination across the healthcare and social care industries or what are sometimes referred to as the human services. We are interested in uncovering what shapes cross-sector interactions between the healthcare and social care industries, specifically related to the provision of services. In addition, we will use the overarching umbrella term consumer to refer to the recipient of the cross-sector service provision with the understanding that numerous terms are used by different sectors, such as patient, client, suspect, student, etc.

The aim of this review is to provide a comprehensive overview of the existing body of evidence related to cross-sector service provision. The overarching research question guiding this umbrella review was: What shapes cross-sector service provision among independent yet interconnected sectors in health and social care? The intent is to uncover what is known about the following:

How is cross-sector service provision conceptualised in the existing literature?

What impacts related to cross-sector service provision and service delivery have been reported? What barriers and facilitators to cross-sector service provision have been identified?

What remains to be known about cross-sector service provision?

## Methodology

**Figure 1:**
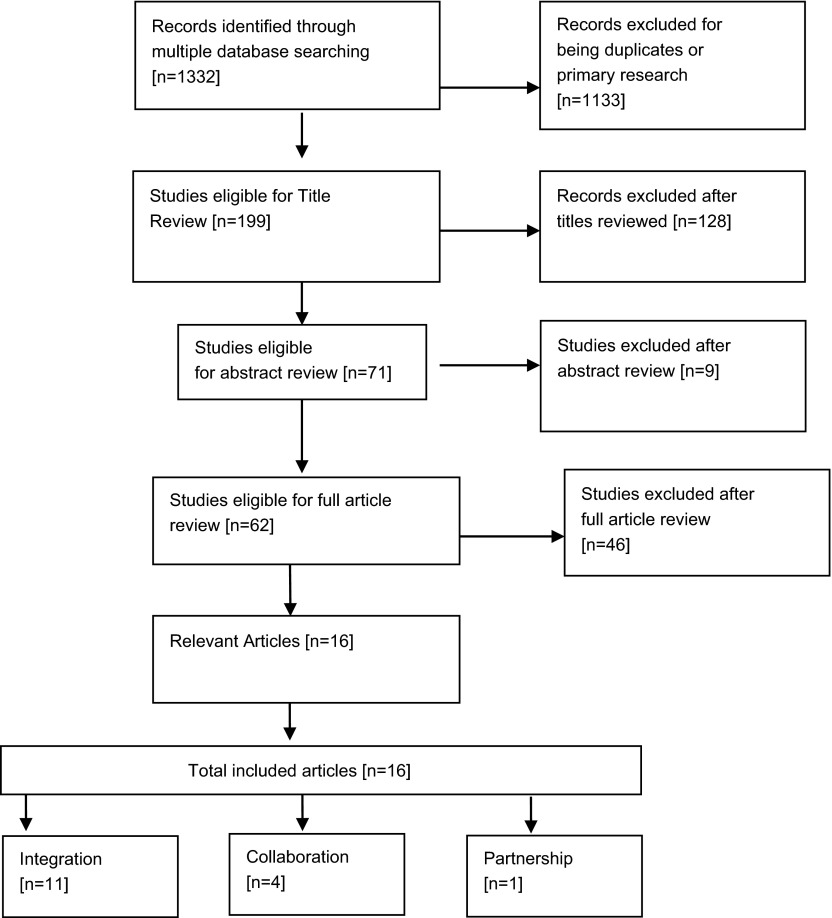
Study selection and exclusion flow diagram.

Initially, the current authors intended to conduct a systematic review of the literature related to cross-sector service provision. At the outset, the search criteria were broad, intended to capture all existing literature in the area. However, in gauging the scope of the literature, it became clear that the pool of evidence was massive and growing rapidly (see Figure 1). Given the extent of the existing literature, it was decided that an overview of reviews would be a more efficient and useful approach. Consequently, the inclusion criteria were narrowed to include only previously conducted systematic reviews. When a plethora of existing literature exists, those who make decisions in health and social care (clinicians, leadership, informed consumers and policy makers) may be overwhelmed trying to determine what evidence to consider when making their decisions [11, p.1, 13, p. 616]. Overarching reviews are becoming a welcomed alternative to traditional reviews as they provide a means of showcasing a wide picture, articulating an overall state of a particular content area [11,12,13,14], or a ‘Friendly Front End’ to massive pools of evidence [13, p. 608]. They also enable a more comprehensive overview of the gaps and inconsistencies that exist and provide direction for future research and practice [13]. The current review aligns with the parameters outlined by the Joanna Briggs Institute [11,12] for conducting *Umbrella Reviews*, more so than the parameters outlined by the Cochrane Collaboration for an *Overview of Reviews* in that we aim to incorporate all types of syntheses (systematic reviews, meta-analyses, narrative reviews, critical reviews and scoping reviews) as opposed to only including previously conducted Cochrane reviews.

### Search methods and scope

Scopus, PubMed and psychInfo databases were searched (see Table 1 for a list of search terms). Original search conducted on November 11, 2014, and replicated on September 6, 2015. One new article was obtained during the September search through PubMed [18]. The following inclusion criteria were adopted to frame the scope of this review of reviews: Review methodology must be explicitly stated, focus on cross-sector service provision or delivery among the health and/or social industries and be published from 2004 onwards. The date range was restricted to focus on a current view of literature and because a review of reviews by Sloper [10] had been previously conducted in a similar area (coordination). Given that we were looking to obtain a comprehensive account of reviews exploring cross-sector service provision, no restrictions were placed on geographical location, intervention type, or typology of published reviews.

**Table 1:** Search terms.


health OR social care, AND partnership, OR collaboration, OR integration, OR joint working, OR coalition, OR alliance, OR interprofessional, AND cross sector, OR bridging, OR multisite, OR inter-sectorial, OR across, AND service provision OR delivery

Reasons for excluding articles included the following: studies were primary research articles and discussion or position articles, not related to direct service provision/delivery; focused on one site; focused on disciplines rather than sectors; related to one professional group only; fell outside of the healthcare and social care fields; methodology was not explicitly stated and reviews of tools that measured partnership but were not studies that explored service provision. Only those studies published in English were included. The search was limited to include only review articles that had been peer reviewed.

Initial search results were restricted to review articles and duplicates were removed. Titles of each article were reviewed against the eligibility criteria and all potentially relevant articles were sent forward. Article abstracts as well as ostensibly relevant full articles were reviewed against the eligibility criteria by authors SW and LM. SW and LM were in agreement in regard to including all 16 articles but initially diverged on whether to include Sloper [10] in the reviewed articles. The authors later agreed not to include Sloper [10] in the reviewed articles but used it as a comparison article to see how the literature has changed over the last 10 years.

A quality appraisal was conducted for all articles using the Joanna Briggs Critical Appraisal Checklist [11] (see Table 2). Reviews such as those by Dowling et al. [15], Loader et al. [16] and Soto et al. [17] were included because we felt that they provided useful information, even though rated on the low side on the Joanna Briggs Quality Appraisal Checklist [11]. Certain data elements were extracted and stored in tabular form (see Tables 1,2,3,4 and 5) using a modified version of Joanna Briggs data extraction form [11]. Included articles were analysed thematically to depict what the literature says about how cross-sector service provision has been conceptualised, what impacts have been reported, what facilitates and precludes cross-sector service provision and future directions for research, policy and practice.

**Table 2:** Quality appraisal checklist scores.

Author	Score	Reject or retain

Butler (2011)	10/11	Retain
Collet (2010)	10/11	Retain
Davies (2011)	10/11	Retain
Donald (2005)	8/11	Retain
Dowling (2004)	5/11	Retain
Fisher (2012)	7/11	Retain
Fleury (2006)	6/11	Retain
Green (2014)	11/11	Retain
Grenfell (2013)	8/11	Retain
Hillier (2010)	8/11	Retain
Howarth (2006)	9/11	Retain
Hussain (2014)	10/11	Retain
Lee (2013)	6/11	Retain
Loader (2008)	1/11	Retain
Soto (2004)	5/11	Retain
Winters (2015)	8/11	Retain

**Table 3:** Characteristics of included studies of cross-sector service provision.

Author	Title	Consumer group	Sectors	Intervention	Primary term	Year range of included studies	Location of authors	Included articles

Butler (2011)	Does integrated care improve treatment for depression?	Individuals with depression	Mental health and primary care	Integrated care planning	Integration	1995–2006	USA	49
Collet (2010)	Efficacy of integrated interventions combining psychiatric care and nursing home care for nursing home residents: A review of the literature	Nursing home clients with mental health concerns	Psychiatric care and nursing homes	Integrated interventions combining both psychiatric care and nursing home care in nursing home residents	Integration	1996–2003	Europe	8
Davies (2011)	A systematic review of integrated working between care homes and health care services	Nursing home patients with primary care needs	Healthcare services and care homes	Interventions designed to develop, promote or facilitate integrated working between care home or nursing home staff and healthcare practitioners	Integration	1998–2008	UK	17
Donald (2005)	Integrated versus non-integrated management and care for clients with co-occurring mental health and substance use disorders: A qualitative systematic review of randomized controlled trials	Mental health and substance abuse	Mental health and substance-use disorder	Integrated approaches are compared with non-integrated approaches for treatment of adults with co-occurring mental health and substance-use disorder	Integration	1993–2001	Australia	10
Dowling (2004)	Conceptualizing successful partnerships	General health and social care	Not described	Not described	Partnership	1999–2003	UK	36
Fisher (2012)	Health and social services integration: A review of concepts and models	Veterans	Veterans health and social care - general	Different approaches to services integration for the needs of veterans	Integration	1993–2008	USA	76
Fleury (2006)	Integrated service networks: The Quebec case	General – not specified	General – not specified	Not mentioned	Integration	1961–2005	Canada	46*
Green (2014)	Cross sector collaboration in Aboriginal and Torres Strait Islander childhood obesity: A systematic integrative review and theory-based synthesis	Indigenous children with disability	Health, education and social services	Inter- and intra- sector collaboration in Aboriginal and Torres Strait Islander childhood disability	Collaboration	2001–2014	Australia	18
Grenfell (2013)	Tuberculosis, injecting drug use and integrated HIV-TB care: A review of the literature	Human Immunodeficiency Virus (HIV), injecting drug user (IDU) with Potential for Tuberculosis (TB)	Health, substance abuse and social services	Governmental or non-governmental health or community-based services providing testing, prevention, treatment or other care for TB or HIV and TB, either directly or by referral.	Integration	1995–2011	UK	87
Hillier (2010)	A systematic review of collaborative models for health and education professionals working in school settings and implications for training	School-aged children	Education and health sectors related to children of school age	Interdisciplinary or multidisciplinary teams and any conclusions drawn about the knowledge or skills required by the professionals to promote these models.	Collaboration	1980–2005	Australia	34
Howarth (2006)	Education needs for integrated care: A literature review	Primary care	Primary care with social care	Education and training initiatives	Integration	1995–2002	UK	25
Hussain (2014)	Integrated models of care for medical inpatients with psychiatric disorders: A systematic review	Medicine in patients with psychiatric concerns	Health and mental health	Integrated models of care where psychiatrists and general medical physicians, either in isolation or in combination with other allied health staff, were integrated within a single team to provide care to an entire inpatient population.	Integration	1997–2010	Canada	4
Lee (2013)	What is needed to deliver collaborative care to address comorbidity more effectively for adults with a severe mental illness?	Mental health –Adults with comorbid concerns	Mental health, employment, forensic, homelessness, housing, physical health and substance abuse	Models that have addressed comorbidities to Severe Mental Illness, to demonstrate key principles needed to promote collaborative care.	Collaboration	1995–2012	Australia	76*
Loader (2008)	Health informatics for older people: A review of ICT facilitated integrated care for older people	Older people with health conditions needing welfare support	Information technology, Computer science and health care (hospitals, clinics, laboratories, surgeries) and social and community agents (housing, voluntary and community groups, social services, carers, community nurses)	Dimensions of care as they were seen to relate to the modernising of adult social care objectives.	Integration	1981–2005	UK	35*
Soto (2004)	Literature on integrated HIV care: A review	HIV and Substance Use Disorder (SUD)	Social services, mental health, and substance abuse	Integrated HIV care models HIV-infected clients and their use of ancillary services and integrated mental health and substance abuse treatment	Integration	1990–2003	USA	47
Winters (2015)	Interprofessional collaboration in mental health crisis response systems: A scoping review	Adult mental health crisis	Mental health, emergency department, police, pharmacy, traditional healers, university campus support	Studies that included an intervention or routine for the specific purpose of improving, measuring or exploring Interprofessional Collaborative Practice	Collaboration	2000–2012	Canada	18

* Indicates data that were not specified by the original author, but determined by the authors of the current study.

**Table 4:** Purpose and main findings of included review articles.

Author	Title	Purpose	Main findings

Butler (2011)	Does Integrated Care Improve Treatment for Depression?	To assess whether the level of integration of provider roles or care process affects clinical outcomes.	Although most trials showed positive effects, the degree of integration was not significantly related to depression outcomes. Integrated care appears to improve depression management in primary care patients, but questions remain about its specific form and implementation.
Collet (2010)	Efficacy of integrated interventions combining psychiatric care and nursing home care for nursing home residents: A review of the literature	Not stated	*N* = 8 (4 randomised controlled studies). Seven studies showed beneficial effects of a comprehensive, integrated multidisciplinary approach combining medical, psychiatric and nursing interventions on severe behavioural problems in nursing home patients. Important elements include a thorough assessment of psychiatric, medical and environmental causes as well as programmes for teaching behavioural management skills to nurses. DCD nursing home patients were found to benefit from short-term mental hospital admission.
Davies (2011)	A systematic review of integrated working between care homes and health care services	To evaluate the different integrated approaches to healthcare services supporting older people in care homes and identify barriers and facilitators to integrated working	Most quantitative studies reported limited effects of the intervention; there was insufficient information to evaluate cost. Facilitators to integrated working included care home managers’ support and protected time for staff training. Studies with the potential for integrated working were longer in duration. Limited evidence about what the outcomes of different approaches to integrated care between health service and care homes might be. The majority of studies only achieved integrated working at the patient level of care and the focus on health service-defined problems and outcome measures did not incorporate the priorities of residents or acknowledge the skills of care home staff
Donald (2005)	Integrated versus non-integrated management and care for clients with co-occurring mental health and substance use disorders: A qualitative systematic review of randomized controlled trials	To examine integrated treatment approaches versus non-integrated treatment approaches for people with co-occurring mental health/substance use disorders in order to investigate whether integrated treatment approaches produce significantly better outcomes on measures of psychiatric symptomatology and/ or reduction in substance use	The findings are equivocal with regard to the superior efficacy of integrated approaches to treatment. Clearly, this is an extremely challenging client group to engage and maintain in intervention research, and the complexity and variability of the problems render control particularly difficult. The lack of available evidence to support the superiority of integration is discussed in relation to these challenges
Dowling (2004)	Conceptualizing successful partnerships	To review literature published in the United Kingdom since 1997 to examine the success of partnerships in the healthcare and social care fields. To discuss the definitional and methodological problems of evaluating success in the context of partnerships before proposing approaches to conceptualising successful partnerships	Research into partnerships has centred heavily on process issues, while much less emphasis has been given to outcome success. If social welfare policy is to be more concerned with improving service delivery and user outcomes than with the internal mechanics of administrative structures and decision making, this is a knowledge gap that urgently needs to be filled
Fisher (2012)	Health and social services integration: A review of concepts and models	The immediate goal of this review of literature is to (a) trace the various definitions and uses of the concept; (b) explain the rationales for services integration; (c) describe how the concept has been utilised theoretically and in practice and provide examples of services integration models; (d) discuss factors that have been found to facilitate or challenge services integration as learned from these applications and (e) inform future development or improvement of policy and related programmes coordinating services and providing outreach to populations in need	Veterans’ services integration models along with inter-organisational relationship (e.g., network) models are common in the literature. Models range from centralised government agency initiatives to less formalised community-based networks of care. Findings from this review of literature may be particularly important to organisations that work with veterans, homeless, chronically ill and aging populations, whose needs often span a number of service areas and who often face multiple delivery systems that heretofore may not have effectively coordinated their services with others
Fleury (2006)	Integrated service networks: The Quebec case	On the basis of a review of publications on services integration and inter-organisational relations and on the Quebec context of healthcare reform, this article aims at generating a greater understanding of the concept of integration and certain underlying issues such as the effectiveness of models	Integrated service networks form of system structuring is one of the main solutions for enhancing efficiency, especially for clientele with complex or chronic health problems. Nevertheless, integrated service networks have lately been highly criticised for their inability to promote better system efficiency, which might be explained by a lack of knowledge in defining models and implementation difficulties. Parameters for organising integrated service networks, either virtual or vertical, have been strongly articulated in response to the lack of knowledge on that notion. The importance of integration strategies and the density of inter-organisational exchange in the network as well as the critical role of governance have been particularly outlined. Finally, information is still lacking on the following topics: effective models and strategies for developing integrated service networks; levels of density and centrality required in a network to achieve better results; clientele’s needs assessment in terms of services and levels of continuity and their influence on network modelling; impact of integrated service network models on system effectiveness, and clientele health and well-being. Impact assessment on integrated services network is central, but the level of reform implementation needs to be evaluated before measuring that impact (the black box effect.) The literature on network implementation and change stresses the importance of investing time and energy in developing tangible strategies to support a reform
Green (2014)	Cross sector collaboration in Aboriginal and Torres Strait Islander childhood obesity: a systematic integrative review and theory-based synthesis	To identify important components involved in inter-and intra-sector collaboration in Aboriginal and Torres Strait Islander childhood disability	Structure of government departments and agencies. The siloed structure of health, education and social service departments and agencies was found to impede service integration and the ability of providers to work collaboratively. Policies collaboration at the level of policy making can address the barriers generated by existing structures of government departments and agencies. Formalised agreements like memoranda of understanding and collaborative frameworks between government sectors can facilitate collaboration at the level of service provision. Communication – Lack of awareness can lead to duplication of resources. Raising awareness of collaborative partnerships through the distribution of educational resources across agencies and services facilitates collaboration. Lack of role clarity and responsibility, ambiguity and lack of role clarity and responsibilities of different providers, agencies and organisations is a key barrier to collaboration. Financial and human resources providing service when resources are limited is a barrier and often are done so ‘on sheer good will’ with staff often working beyond their normal hours. Service delivery setting: The effectiveness of a collaborative programme is influenced by the setting in which it is delivered. Relationships: A key facilitator to collaboration at this level is the coordinator or linking role. The appointment of a person external to the services or agencies involved whose role is to link the different players and act as a trainer, motivator and sustainer can be important to a collaborative interdisciplinary approach. Inter- and intra-professional learning: The modelling of inter- and intra-professional collaboration by clinical educators from different disciplines for university students on placement has been reported to facilitate a well-coordinated and holistic approach to learning
Grenfell (2013)	Tuberculosis, injecting drug use and integrated HIV-TB care: A review of the literature	This study builds on a recent review of tuberculosis among people who use drugs (Deiss et al., 2009) but focuses specifically on persons who inject drugs, a socially marginalised group with complex treatment needs. Specifically, to (1) describe the prevalence, incidence and risk factors for tuberculosis, Multidrug Resistance (MDR)-tuberculosis, and HIV-tuberculosis and HCV– HIV tuberculosis co-infections among persons who inject drugs and (2) identify models of tuberculosis and HIV tuberculosis care for persons who inject drugs	Latent tuberculosis infection prevalence was high and active disease more common among HIV-positive persons who inject drugs. Data on multidrug-resistant tuberculosis and coinfections among persons who inject drugs were scarce. Models of tuberculosis care fell into six categories: screening and prevention within HIV-risk studies; prevention at TB clinics; screening and prevention within needle-and-syringe-exchange and drug treatment programmes; pharmacy-based tuberculosis treatment; tuberculosis service-led care with harm reduction/drug treatment programmes; and TB treatment within drug treatment programmes. Co-location with needle-and-syringe-exchange and opioid substitution therapy, combined with incentives, consistently improved screening and prevention uptake. Small-scale combined TB treatment and opioid substitution therapy achieved good adherence in diverse settings. Successful interventions involved collaboration across services, a client-centred approach and provision of social care. Grey literature highlighted key components: co-located services, provision of drug treatment, multidisciplinary staff training; and remaining barriers: staffing inefficiencies, inadequate funding, police interference, and limited opioid substitution therapy availability. Integration with drug treatment improves persons who inject drugs engagement in TB services but there is a need to document approaches to HIV-TB care, improve surveillance of TB and co-infections among persons who inject drugs and advocate for improved opioid substitution therapy availability
Hillier (2010)	A systematic review of collaborative models for health and education professionals working in school settings and implications for training	Search of the literature to reveal the rudimentary state of the art in conceptualising, measuring and demonstrating the success of partnerships	Models of interaction and teamwork are well described, but not necessarily well evaluated, in the intersection between schools and health agencies. They include a spectrum from consultative to collaborative and interactive teaming. It is suggested that professionals may not be adequately skilled in, or knowledgeable about, team work processes or the unique roles each group can play in collaborations around the health needs of school children
Howarth (2006)	Education needs for integrated care: A literature review	To identify and critically appraise the evidence base in relation to education needed to support future workforce development within a primary care and to promote the effective delivery of integrated health and social care services	Six themes were identified which indicate essential elements needed for integrated care. The need for effective communication between professional groups within teams and an emphasis on role awareness are central to the success of integrated services. In addition, education about the importance of partnership working and the need for professionals to develop skills in relation to practice development and leadership through professional and personal development are needed to support integrated working. Education that embeds essential attributes to integrated working is needed to advance nursing practice for interprofessional working
Hussain (2014)	Integrated models of care for medical inpatients with psychiatric disorders: A systematic review	To review the different models of integrated models of care for medical inpatients with psychiatric disorders and to examine the effects of integrated models of cares on mental health, medical and health service outcomes when compared with standard models of care	In two studies, integrated models of care improved psychiatric symptoms compared with those admitted to a general medical service. Two studies demonstrated reductions in length of stay with integrated models of cares compared with usual care. One study reported an improvement in functional outcomes and a decreased likelihood of long-term care admission associated with integrated models of care when compared with usual care. There is preliminary evidence that integrated models of care may improve a number of outcomes for medical inpatients with psychiatric disorders
Lee (2013)	What is needed to deliver collaborative care to address comorbidity more effectively for adults with a severe mental illness?	To identify Australian collaborative care models for adults with a severe mental illness, with a particular emphasis on models that have addressed comorbidities to a severe mental illness, to demonstrate key principles needed to promote collaborative care	A number of nationally implemented and local examples of collaborative care models were identified that have successfully delivered enhanced integration of care between clinical and non-clinical services. Several key principles for effective collaboration were also identified. Governmental and organisational promotion of and incentives for cross-sector collaboration is needed along with education for staff about comorbidity and the capacity of cross-sector agencies to work in collaboration to support shared clients. Enhanced communication has been achieved through mechanisms such as the co-location of staff from different agencies to enhance sharing of expertise and interagency continuity of care, shared treatment plans and client records and shared case review meetings. Promoting a ‘housing first approach’ with cross-sector services collaborating to stabilise housing as the basis for sustained clinical engagement has also been successful
Loader (2008)	Health informatics for older people: A review of information and communications technology facilitated integrated care for older people	To find examples of good practice and any evidence to support the high expectations and confidence in information and communications technology to effectively address the challenges of healthcare and social care of older people	The aspiration of information and communications technologies to reconcile competing models of care also foregrounds the importance of recognising that information and communications technologies are designed and diffused within a particular social context that can either stimulate its adoption or make it redundant. The fastest broadband network connection will be of little use if healthcare and social care professionals are not prepared to share information with each other, let alone allow access to older people wishing to participate in decisions about their care. Similarly, the most accessible website will be seldom used by older people if its information content is not perceived as relevant to the life experiences of the user. Thus, while information and communications technologies may be regarded as important tools for enabling the ‘modernisation’ objectives to be achieved, their effectiveness is crucially shaped by the outcome of debates about those objectives themselves. Information and communications technologies cannot be viewed as a means to reconcile such policy contradictions. Such confused rhetoric is only likely to produce expensive and ineffective health informatics outcomes. The contradictions will merely be encoded into the system. Despite the repeated policy claims for health informatics to facilitate integrated person-centred health and social care, there is little evidence in the literature review considered here that it has been realised
Soto (2004)	Literature on integrated HIVcare: A review	It presents the findings related to integrated HIV care models, the needs of HIV-infected clients and their use of ancillary services, and integrated mental health and substance-abuse treatment, as well as descriptions of innovative integrated HIV care programmes. With the goal of providing useful information to HIV service providers, programme managers, and policy makers, these findings are discussed, and directions for future research are offered	The few evaluations of integrated models tended to focus on measurements of engagement and retention in medical care, and their findings indicated an association between integrated HIV care and increased service utilisation. The majority of reviewed articles described integrated models operating in the field and various aspects of implementation and sustainability. Overall, they supported use of a wide range of primary and ancillary services delivered by a multidisciplinary team that employs a ‘biopsychosocial’ approach. Despite the lack of scientific knowledge regarding the effects of integrated HIV care, those wanting to optimise treatment for patients with multiple interacting disorders can gain useful and practical knowledge from this literature
Winters (2015)	Interprofessional collaboration in mental health crisis response systems: a scoping review	To rapidly map key contributions to knowledge, especially in areas that are complex or have not yet been reviewed comprehensively, to summarise and disseminate research findings and to identify gaps in the existing literature related to interprofessional collaboration in mental health crisis response systems	Support for interprofessional collaboration, quest for improved care delivery system, merging distinct visions of care and challenges to interprofessional collaboration. Lack of conceptual clarity, absent client perspectives, unequal representation across sectors and a young and emergent body of literature were found. Key concepts need better conceptualisation, and further empirical research is needed

**Table 5:** Frequency of terms used interchangeably by authors of included studies.

	Butler	Collet	Davies	Donald	Dowling	Fisher	Fleury	Green	Grenfell	Hillier	Howarth	Lee	Loader	Hussain	Soto	Winters	Number of terms used

Alliance						▪	▪										2
Client centred									▪	▪					▪		3
Collaborating/ion	▪	▪	▪	▪	▪	▪	▪	▪ *	▪	▪ *	▪	▪ *		▪	▪	▪ *	15
Consolidating/ion				▪		▪	▪										3
Coordinating/ion	▪			▪	▪	▪	▪	▪	▪	▪	▪	▪				▪	11
Integrating/ion	▪*	▪ *	▪ *	▪ *	▪	▪ *	▪ *	▪	▪ *	▪	▪ *	▪	▪ *	▪ *	▪ *	▪	16
Interdisciplinary						▪	▪	▪		▪	▪					▪	6
Inter-organisational						▪	▪	▪									3
Inter-agency					▪	▪		▪				▪					4
Inter-sectorial						▪	▪										2
Inter-professional								▪		▪	▪					▪	4
Joint ventures/ working/ initiative /care			▪		▪	▪				▪	▪	▪	▪	▪			8
Multi-agency										▪			▪				2
Multidisciplinary		▪	▪				▪	▪	▪	▪	▪	▪	▪	▪	▪	▪	12
Multi-organisational						▪	▪										2
Multi-professional				▪			▪				▪						3
Partnership			▪		▪ *	▪	▪	▪	▪		▪	▪	▪			▪	10
Spoke/Case management /coordination	▪	▪		▪		▪	▪		▪	▪		▪		▪	▪	▪	11
Team	▪	▪	▪	▪	▪	▪	▪			▪	▪	▪			▪	▪	12
Teamwork			▪							▪	▪	▪		▪			5
Trans-disciplinary										▪							1
Trans-organisational						▪											1
Vertical integration							▪								▪		2
Virtual integration					▪		▪										2
Terms used per article	5	5	7	7	7	15	16	9	7	13	11	10	6	6	7	9	140

*Indicates the primary term adopted by the authors.

## Findings

The included reviews were heterogeneous and a meta-analysis of quantitative findings was not possible; therefore, the available findings are presented in narrative form. Sixteen articles were reviewed (see Fig. 1 for the study selection and exclusion flow diagram and Table. Table 3 for the characteristics of included studies).

The included review articles were published between 2004 and 2015, and the time period of the individual primary articles represented in the reviews spans 1961–2012. Authors were from Europe (*n* = 6), Australia (*n* = 4), Canada (*n* = 3) and the United States (*n* = 3). Analysis of where the individual studies were conducted could not be determined because many authors did not report this information. Consumer groups included school-aged children with health concerns, adults with comorbidity concerns (mental health with various other sectors), adults living with a disability, veterans, nursing/care home patients, persons living with HIV and persons accessing primary care in general. Service type provided in the included reviews spanned acute, primary and community care. The number of articles included in each review ranged from 4 to 87.

Authors used various terms to describe cross-sector service provision, which will be discussed further in the following section; however, the primary terms^2^ used by authors in the included reviews are Integration (*n* = 11), Collaboration (*n* = 4) and Partnership (*n* = 1). Type of primary research study included in the reviews could not be determined because the authors did not consistently report this information. Of the articles that did describe study type, there was a broad range. Six reviews included findings from randomised control trials [19,20,21,22,23,24], while others reported on a mix of quasi-experimental design studies, qualitative studies, basic descriptions of models (either in use or hypothetical) and evaluation outcomes. Overall, a number of the authors of the review articles commented on the lack of reported outcomes and evaluations of cross-sector service provision arrangements [15,16,25,26,27]. Lack of evaluation will be discussed later in this article (see Table 4 for an overview of the purpose and main findings of the included studies).

The previously conducted review of reviews by Sloper [10] published in 2004 will be referenced in this article as baseline knowledge to see how the field has evolved over the last 10 years. Sloper [10] explored what they refer to as coordinated multi-agency working (which the author also calls Joint Working and Collaboration). In a sense, our review offers both an overview of the massive body of evidence related to cross-sector service provision and also updates the findings since Sloper’s [10] review was conducted. Throughout this article, we will refer to similarities and differences and indicate gaps that remain, as well as highlight novel areas to consider in moving the field of research forward.

### How is cross-sector service provision conceptualized in the existing literature?

In this section, we discuss the emergent terminology that appears to inform the overarching concept of cross-sector service provision. Determining how the authors conceptualised cross-sector service provision was challenging. Numerous terms are used interchangeably and with great frequency in the included articles (see Table 5 for the frequency breakdown).

Cross-sector service provision appears to be informed by a number of separate bodies of literature. The current findings suggest that three concepts primarily inform the cross-sector service provision included in the studies: Integration, Collaboration and Partnership. Integration is the most commonly used term and is used in each of the 16 included articles. Collaboration is the second most used term, found in all but one of the included articles. Interestingly, multidisciplineary is used in 12 of the 16 articles despite none of the authors adopting it as a primary term. Authors in the included studies also frequently use team and teamwork, as well as case management. Only half (*n* = 8) of the authors of the included articles articulate their interpretation of the primary terms they adopt. Authors who do include a definition use a number of different terms as though they are synonymous with the primary term. The range of different terms used ranges from 5 to 16 terms, with the average being 9, and midpoint being seven terms used per article. On average, authors use nine different terms, often synonymously, when referring to cross-sector service provision, yet it can be argued that each of those terms are not synonymous with one another. There is also variation in how the authors define the same primary term (see Table 6 for a breakdown of definitions articulated by the authors). Even when the same primary term is used across different articles, rarely do the authors define the terms in the same way. In addition, elements of the definitions overlap regardless of the primary term. Common elements from the various definitions include independent sectors working together to improve care, focusing on the consumer and understanding that consumer needs are complex [4,15,17,18,19,21,22,24,28].

Integration and collaboration literatures have begun to discuss cross-sector service provision as occurring at different levels but differences exist in how these levels are conceptualised. As Davies et al. [21], Fisher and Elnitsky [25] and Green et al. [18] outline, cross-sector service provision can occur at three levels. Davies et al. [21] and Fisher and Elnitsky [25] label the levels as micro/patient, meso/organisational or macro/strategic. With slight variation, Green et al. [18] uses the following distinctions for levels: macro/government, exo/organisational and meso/provider levels. Although similarly discussed, there are variations in how the terms are used in the literature. Other authors who adopt different primary terms (Integration, Collaboration and Partnership) do not formally make the distinction between the levels, but do speak to elements required for effective cross-sector service provision that are similar to the levels outlined above.

**Table 6:** Definitions of primary concept used by authors of included studies.

Author	Primary term	Definition provided

Butler (2011)	Integration	At the simplest level, integrated mental and physical health care occurs when mental health specialty and general medical care clinicians work together to address both the physical and mental health needs of their patients. Models of integrated care, sometimes called collaborative care, vary widely, but most include more than merely enhanced coordination of or communication between the clinicians responsible for the mental and physical health needs of their patients. Indeed, attempts to integrate provider roles emphasise parity and mutual respect for the two health components. At the same time, they include efforts to improve the process of care using evidence-based standards of care.
Collet (2010)	Integration	Not defined
Davies (2011)	Integration	Integration of service provision can be defined as ‘a single system of needs assessment, commissioning and/or service provision that aims to promote alignment and collaboration between the cure and care sectors (Rosen & Ham, 2008). There are different levels of integration between healthcare services (Kodner & Spreeuwenberg, 2002). In the context of integrated working with care homes, these can be summarised as: Patient/micro-level close collaboration between different healthcare professionals and care home staff, e.g. for the benefit of individual patients. Organisational/meso-level organisational or clinical structures and processes designed to enable teams and/or organisations to work collaboratively towards common goals (e.g. integrated health and social care teams). Strategic/macro-level integration of structures and processes that link organisations and support shared strategic planning and development, e.g. when healthcare services jointly fund initiatives in care homes (Bond, Gregson, & Atkinson, 1989 and The British Geriatrics Society, 1999).
Donald (2005)	Integration	There is considerable diversity concerning the definition of integration, and the extent of integration varies enormously across different studies and settings. For example, it is used to refer to treatment provided both by multi-professional teams and by individual providers. In general, integrated approaches refer to those where both the mental health disorder and the addictive disorder are treated simultaneously. Typically, this is regarded as requiring the treatment to take place within the same service by the same clinician. The nature of the integrated treatment should also be considered. If integration merely involves augmentation through the addition of either a standard mental health treatment component or a standard drug and alcohol treatment component, then it may be argued that this is not truly integrated. Rather it may be that an integrated treatment would directly acknowledge and address the presence of the comorbidity in terms of the tailoring of the treatment to the current status of the person and would treat the co-occurring nature of the disorders, which may involve making adjustment in one treatment to take account of the other.
Dowling (2004)	Partnership	For the purposes of the present article, the authors adopted the Audit Commission’s (1998) definition of partnership as a joint working arrangement where partners are otherwise independent bodies cooperating to achieve a common goal; this may involve the creation of new organisational structures or processes to plan and implement a joint programme, as well as sharing relevant information, risks and rewards. This definition is compatible with a wider range of terms than ‘partnership’, including similar terms such as ‘cooperation’ and ‘collaboration’.
Fisher (2012)	Integration	Not defined
Fleury (2006)	Integration	To paraphrase Leutz (1999) the term integration has put forward a large number of models concerning the organisation of services or types of intervention. All refer to ‘anything from the closer coordination of clinical care for individuals to the formation of managed care organisations that either own or contract for a wide range of medical and social support services’.
Green (2014)	Collaboration	Not defined
Grenfell (2013)	Integration	Not defined
Hillier (2010)	Collaboration	Not defined
Howarth (2006)	Integration	Not defined
Hussain (2014)	Integration	Collaborative or integrated mental health care has been defined as care delivered by general medical physicians working with psychiatrists and other allied health professionals to provide complementary services, patient education and management to improve mental health outcomes (Katon, Von Korff, & Lin, 1995). Integrated models of care are patient-centred, and they not only involve the psychiatrist as a consultant with co-location of psychiatric and medical services but also involve a shared responsibility for the care of all patients within a service.
Lee (2013)	Collaboration	Not defined
Loader (2008)	Integration	Not defined
Soto (2004)	Integration	For the purpose of this review, we offer the following working definition: Integrated HIV care combines HIV primary care with mental health and substance-abuse services into a single coordinated treatment programme that simultaneously, rather than in parallel or sequential fashion, addresses the clinical complexities associated with having multiple needs and conditions.
Winters (2015)	Collaboration	Craven and Bland (2006) ‘involving providers from different specialties, disciplines, or sectors working together to offer complementary services and mutual support, to ensure that individuals receive the most appropriate service from the most appropriate provider in the most suitable location, as quickly as necessary, and with minimal obstacles’.

Craven MA, Bland R. Better practices in collaborative mental health care: an analysis of the evidence base. Can J Psychiatry Revue Canadienne De Psychiatrie 2006; 51: 7S–72S. Leutz WN. Five laws for integrating medical and social services: lessons from the United States and the United Kingdom. Milbank Quart 1999; 77: 77–110. Rosen R, Ham C: Integrated Care: Lessons from Evidence and Experience. The Nuffield Trust for Research and Policy Studies in Health Services; 2008. Kodner DL, Spreeuwenberg C: Integrated care: meaning, logic, applications, and implications – a discussion paper. International journal of integrated care 2002, 2[1]: 1–6. Bond J, Gregson BA, Atkinson A: Measurement of Outcomes within a Multicentred Randomized Controlled Trial in the Evaluation of the Experimental NHS Nursing Homes. Age and Ageing 1989, 18: 292–302. British Geriatrics Society: The Teaching Care Home – an option for professional training. Proceedings of a Joint BGS and RSAS AgeCare Conference held in February 1999 [http://www.bgs.org.uk/PDF%20Downloads/teaching_care_homes.pdf], accessed 080311. Katon W, VonKorff M, Lin E, et al: Collaborative management to achieve treatment guidelines Impact on depression inprimarycare. JAmMedAssoc1995; 273: 1026–1031. Audit Commission [1998] A Fruitful Partnership: Effective Partnership Working. Audit Commission, London.

Although not all of the included reviews specify a theoretical framework, some authors name theories that might be helpful in working towards bringing greater conceptual clarity to cross-sector service provision. Some of the theories mentioned include federalism theory, governance theory, interorganisational theory, intersectoral theory, institutional change theory, innovation theory, public choice theory, humanistic theory, boundary theory [25] and professional socialisation theory [29]. However, how these theories specifically align with the included articles was not specified.

The findings above indicate the need to clearly identify what is meant by cross-sector service provision and to pay particular attention to the differences between some of the more commonly used terms such as integration, collaboration, partnership and coordination. As described above, these terms have different meanings and should not be used synonymously As Kodner [2, p. 4] states, ‘as is often the case with nascent fields, especially those with a strongly multidimensional character, the defining concepts and boundaries lack specificity and clarity. Thus, the definitions that are commonly used tend to be vague and confusing. This makes it difficult to develop the knowledge base essential to refine and move the field ahead’. More consideration of terminology is needed.

### What impacts related to cross-sector service provision and service delivery have been reported?

The authors of the included reviews strongly support the need to collaborate across sectors to provide more comprehensive, faster and more appropriate care to consumers [15,16,19,21,22,23,24,28,29]. Despite the strong support for cross-sector service provision and many articles reporting positive impacts related to processes, only four included reviews report positive outcomes related to cross-sector service provision. Seven articles in Collet et al. [20] indicate the beneficial effect of cross-sector service provision combining medical, psychiatric and nursing interventions for severe behavioural problems in nursing home patients needing both psychiatric and nursing care. Hussain & Seitz [24] found that integrated models of care (provided by general medical physicians, psychiatrists, and other allied health professionals) are associated with improvements in psychiatric care and that length of stay and re-admission rates of long-term placements may be reduced by integrated models of cares. The authors conclude that there is some preliminary evidence to suggest that integrated models of cares are helpful in improving care for this complex population. Dowling et al. [15] found that cross-sector service provision leads to improvements in the accessibility of services to users; more equitable distribution of services; the efficiency, effectiveness or quality of services delivered through partnerships; improved experiences of staff and informal care givers; improved health status, quality of life or well-being experienced by people using services; and reductions in otherwise likely deteriorations in their health. The majority of the studies reviewed by Butler et al. [19] show significant benefit with regard to treatment response and remission, but only one model shows consistent benefits in terms of improvements in symptom severity. All other included reviews conclude that before any claims to positive outcomes related to cross-sector service provision are possible, further research is needed.

### What barriers and facilitators to cross-sector service provision have been identified?

#### Evolving best practices – consumer centred

Almost half of the included studies stress the importance of placing the consumer at the centre of the cross-sector service provision arrangement [4,17,23,27,29]. This variously involves making sure that consumers are centrally involved in care provision and that their voice is present during decision making [23,27]; establishing trust and ensuring that the consumer’s goals are met [27]; establishing mechanisms for communication across sectors in the event that the consumer’s needs rapidly change [27] and to improve continuity of care [27]. Notably, almost all authors discuss the glaring gap of the missing consumer perspective in all levels of service provision: planning, delivery, policy and research. This will be discussed further in the next section. The general consensus is that taking a consumer-centred approach facilitates cross-sector service provision.

#### Towards a shared vision of care – perceived need*,* commitment and involvement

Striving for a shared vision of care across sectors is mentioned as integral to the success of cross-sector service provision by a number of authors [4,15,25,27,29]. A number of authors suggest that for cross-sector service provision arrangements to be successful, there must first be a perceived need for the arrangement [15,26,27,29] and commitment from all sectors [26,27,29]. Authors stress the importance of involving staff early on in the conceptualisation phase [4] and in an on-going and iterative manner for the duration of the cross-sector service provision arrangement [4,26]. Clarity of goals and purpose are seen as important by a number of authors [15,25,29]. Furthermore, a number of authors suggest that goals of the cross-sector service provision are best developed in a cooperative and coordinated manner [26,27,29]. Decision making that occurs in a collaborative and shared manner is also reported to facilitate cross-sector service provision [26,27]. Winters et al. [4] suggest that devoting time to work through differences as they emerge between sectors is important for ensuring that all parties align with a shared vision for the cross-sector service provision arrangement. Sloper [10] highlights similar findings and notes that if the reverse (lack of perceived need and shared vision) is found to be the case, it acts as a barrier to the success of cross-sector service provision.

Many authors mention that equality across sectors involved in cross-sector service provision plays an important role in providing better care [4,17,21,26,29]. In particular, Soto et al. [17] highlight that arranging the team members in a non-hierarchical way facilitates a high level of collaboration [17]. Winters et al. [4] report that at times included studies would regard one sector as the knower and one as the learner, which could create tension between two sectors. Similarly, Davies et al. [21] report that in all the studies included in their review, healthcare staff, rather than home care staff, led or conducted the programmes. Many home care staff in their studies reported feeling like their knowledge and views were not valued. Equal participation across sectors is therefore viewed to be important for achieving success related to cross-sector service provision.

#### Leadership

Effective leadership is considered to be an integral element of cross-sector service provision [15,17,21,25,27,28]. Sloper [10] indicates that appropriate leadership, if present, is a facilitator to cross-sector service provision and, if lacking, is a barrier in many of the articles they reviewed. Buy-in, on-going support and consistent involvement by leadership are viewed as mechanisms to challenge ways of thinking that preclude cross-sector service provision facilitation [17,21,25,27]. Moreover, suitable leadership is reported to promote the inclusion of approaches that facilitate cross-sector service provision into everyday practice [27]. Fleury [28] indicates that collective leadership, meaning the involvement of all levels of governance – structural, tactical and operational – is necessary to support coherent and integrated support for cross-sector service provision.

#### Service provision across the boundaries

Given that sectors involved in cross-sector service provision are rarely governed under the same body or are socialised differently based on the cultures of their workplaces, providing a service across boundaries can be challenging. Communication (or its absence) is frequently identified as a facilitator or barrier to providing effective cross-sector service provision [4,17,18,26,27]. Many sectors have their own language or jargon [4,26], and as Hillier et al. [26] state, attention is needed to ensure that sectors are not disempowering one another with their use of jargon. Hillier et al. stress that open communication among team members should be fostered and encouraged [26]. Other scholars recommend the implementation of mechanisms to enhance regular and direct communication across sectors such as developing shared or agreed-upon protocols, procedures, service agreements or memoranda of under-standing [17,18,25,27]. Lee et al. [27], Soto et al. [17] and Green et al. [18] state that introducing specific and well-defined protocols and partnership agreements related to performing the intervention of interest are important for clarifying expectations of those involved and for ensuring accountability. Lee et al. [27] also highlight joint or shared treatment planning across sectors as a means of underpinning a model’s success.

Sharing information across sectors is a complex issue requiring deliberate attention from all sectors involved. A number of authors point to the challenges experienced when sectors have different rules related to consumer confidentiality or sharing consumer information [17,26,27]. Lee et al. [27] found that some partners were able to share information freely and that the sharing of information improved over time for those who originally experienced challenges.

On a similar vein, nine reviews speak to the value of having someone in an expert/specialised role or taking on a coordinating function [4,17,18,19,21,25,26,27,28]. Hillier et al. [26] mention that viewing all team members as equal but assigning a leadership role to the individual with the greatest expertise results in greater team functioning and cohesion, as well as promotes an equal distribution of leadership responsibilities. A number of authors note the benefits of having someone in a coordinator, linking, case manager, transition support worker or boundary spanner role [4,17,18,19,27,28]. Lee et al. [27] state that these roles improve client engagement and satisfaction rates. Issues of language use, jargon, confidentiality, agreed-upon documents and coordinator roles are reported to enhance the success of cross-sector service provision.

#### Adequately resourcing cross-sector service provision

Committing adequate resources across sectors that are attempting to offer cross-sector service provision is a critical feature frequently identified in the included reviews [4,18,21,23,25,27]. Funding can be challenging to navigate when sectors retain most or all of their independence from one another [4,21,25,27]. In cross-sector service provision arrangements where the programme is jointly funded, this issue appears to be reduced [21]. However, a number of reviews noted that when there are hard divides between sectors, ensuring that the service provision is funded equally by all involved can be difficult [4,25,27]. Fisher and Elnitsky [25] discuss creative ways of sharing costs when categorical (sector-specific) funding is provided. The authors highlight blended funding and the ‘medical home’ approach as a means of navigating categorical funding arrangements. The medical home approach provides patients with a range of services and blended funding often occurs through fund matching from different sources. Fisher and Elnitsky [25] go on to warn that adopting this approach must be done flexibly given the changing needs of consumers. Lee et al. [27] discuss a funding arrangement where one government department oversaw funding for two sectors with the expectation that care be provided in partnership. Green et al. [18, p. 10] found that often service provision occurred ‘on sheer good will’ where staff worked beyond their normal hours to provide the service. Dedicated time [18,21], appropriate infrastructure, space [4,17] and adequate staffing [4,18,23] are also viewed as critical to the sustainment of cross-sector service provision.

Resources allocated to evaluation and monitoring are noted as critical to ensuring that cross-sector service provision produces the desired effect [15]. The element of evaluation, particularly in regard to outcomes, is reported as largely missing from most of the included studies in the review articles. This will be discussed further later on in this section.

#### Developing novel arrangements or fostering existing relationships

For cross-sector service provision to be implemented, slight to significant changes to standard practice are needed [21,27,28,29]. However, two reviews [27] mention that often when cross-sector service provision arrangements parallel a pre-existing relationship with a history of shared service provision between two or more sectors, they are more successful, rather than when novel partnerships are established. Others stress that the introduction of any model requires that it be moulded to the context in which it will be delivered [23,28], taking into account the unique needs of the consumer group [23]. In Fleury’s [28, p. 162] extensive review of the conceptualisation of integrated service networks, the authors indicate that ‘the networks that have reached a higher level of density, meaning a better integration between organizations, have developed the most formalized ties between organizations through various integration strategies at the structural, functional/ administrative, and clinical levels’. Davies et al. [21] similarly state that formal structures may need to be in place for cross-sector service provision to be successful. Consideration must be given to attempting to align the cross-sector service provision with an existing relationship or devoting time to align novel cross-sector service provision arrangements with the context in which they will be delivered.

#### Strengthening connections between sectors

As Hillier et al. [26] conclude, team building work is one of the biggest predictors of success and this notion was shared by Howarth et al. [29] who stress that the need for team working skills is necessary for strong cross-sector service provision. The stronger the linkages are between the sectors involved, the more successful the cross-sector service provision arrangements are thought to be [17,18]. Cross-training, learning together or increasing knowledge about the other sector is mentioned by a number of authors as a means to strengthen the connection between sectors [7,18,21,27,29]. But again, Davies et al. [21] note that being able to access training requires dedicated resources (time and funding) for such activities and that all levels of staff need to be encouraged to participate in the training.

Role clarification is another area that is mentioned profusely in bodies of literature related to integration, collaboration, partnership and coordination. Findings from this umbrella review further support the notion that clarification of roles is critical to the success of cross-sector service provision [4,18,26,27,29]. Hillier et al. [26] mention that clarifying roles through observing each other’s work contributes to the success of the service delivery. Howarth et al. [29] found that negotiating roles removed professional tribalism and turf war issues. These findings are in line with Sloper’s [10] review where the authors conclude that clearly defined roles ensure that everyone knows what was expected of them. Similar to Sloper’s [10] findings, the degree to which members from each sector respect and trust the members of the other sector shape the success of the cross-sector service provision [15,27,29] and can be promoted by joint training.

Opportunities to meet regularly [4,18,25,27,29], face to face or by phone, or being co-located [4,18,27] are identified as helping to build stronger connections between sectors involved in cross-sector service provision. Reasons for coming together include consulting on a case [4,27], having regular steering committee meetings [27] or to participating in a community of practice [25]. A number of authors of included reviews state that having opportunities to engage with members from other sectors is necessary for success and parallel the findings in Sloper [10].

Contrarily, the constant mutation of services and/or the expansion of roles [29], high staff turnover [18,27], different professional ideologies [4] and turf wars [17,29] are identified as posing challenges to increasing connections across sectors. Green et al. [18] mention the negative impact racism and historical trauma have on cross-sector service provision and this is similar to the discussion in Sloper [10] that relates to the negative impact stereotypes have on staff building strong connections with one another. However, other authors did not mention these notions. Future research should explore these concepts in more depth.

### What remains to be known about cross-sector service provision?

#### Absent consumer voice notable

The main reason for engaging in cross-sector service provision is to improve the care provided to consumers, but what is strikingly absent from much of the included literature is the voice of the consumer [4,19,21,26,28]. Hillier et al. [26] indicate that the bulk of the literature included in their review is from the perspective of the expert opinion, with the values and preferences of the consumer left unreported. Winters et al. [4] similarly state that the consumer perspective is relatively absent and that consumer-related outcomes were reported from the perspective of the caregivers. Only one study in their review reported outcomes from the consumer’s perspective [4]. Numerous authors are strongly calling for more research related to consumer outcomes [4,19,21,26,28]. Including this perspective is critical to ensuring that cross-sector service provisions are provided in a way that meets the needs of the consumer [4].

#### Lack of published evaluation findings and outcomes

Perhaps most notable, all but one of the authors of the included reviews writes about the lack of evaluation and outcomes [4,15,17,19,20,21,22,23,24,25,26,27,28,29]. As Hillier et al. [26] note, models of teamwork are well described but not well evaluated. Making firm recommendations about cross-sector service provision is challenging without evidence [4,15,17,20,21,22,23,24,25,26,27,28,29]. Unfortunately, the findings from the current review strongly align with those found 10 years previously by Sloper [10] indicating that little movement in the way of evaluation and outcome measurement has occurred. Almost all authors in this review implore researchers to include evaluative components in future research related to cross-sector service provision in order to demonstrate effectiveness [4,15,17,20,21,22,23,24,25,26,27,28,29]. Fisher & Elnitsky [25] suggest that evaluations should occur early on, and Lee et al. [27] suggest that they should occur alongside service innovation to measure the success of services integration. Dowling et al. [15] posit that perhaps this paucity is related to the extended time frames and complexity of measuring outcomes. The authors also stress that evaluating processes, although more straightforward to measure, may only be relevant for the duration of the partnership [15]. They conclude that these difficulties may contribute to placing misleading evidence in our grasp. More attention to evaluating cross-sector service provision and determining outcomes is necessary.

## Discussion

The findings from this umbrella review parallel, in many ways, those found by the 2004 review of reviews conducted by Sloper [10]. A more disappointing similarity between the current review and that of Sloper [10] was that even though authors have long-concluded their studies with a plea for more research on outcomes related to the effectiveness of cross-sector service provision, to date very few studies report effectiveness outcomes. Even if reported, the outcomes related to effectiveness were rarely positive. Again, many reviews found that the bulk of the evidence is still related to descriptions or findings related to the process of cross-sector service provision, but at present there is minimal evidence related to the outcomes of cross-sector service provision.

There is much overlap between the supposedly distinct bodies of literature drawn on to inform the current view of cross-sector service provision. The first issue at hand is the lack of conceptual clarity in the literature related to cross-sector service provision. Immediate work is needed to ensure that the emerging body of literature facilitates a dialogue among researchers, policy makers, service providers and consumers regarding what cross-sector service provision entails and its possible benefits and drawbacks. The current authors proposed a working definition of cross-sector service provision as independent, yet interconnected sectors working together to better meet the needs of consumers and improve the quality and effectiveness of service provision – in the hopes that it will spur dialogue and debate needed to progress the field and to ensure coherence in service delivery planning, provision and sustainment.

The terms used interchangeably carry similar but not identical meanings; therefore, care must be taken to ensure that researchers and decision makers understand the nuanced differences in the terms they are using. Moreover, attention is required to exploring cross-sector service provision at intersection of care transition points, as opposed to at the individual provider level. Much remains to be known about what shapes cross-sector service provision and the outcomes that result from these arrangements. The current authors could not easily discern from the included literature why any of the included reviews adopted one primary term over another. Of the authors who did delineate how they conceptualised the central term, there was substantial overlap between their definition and that of other authors’ definition of different central terms.

### Future research implications

Significant time and attention must be given to conceptualising how all sectors involved in the provision of services make sense of the arrangement. This includes things like developing a shared vision, strategies to facilitate communication and awareness of what to do about power differentials. Moreover, additional work is needed to determine what occurs at the boundary between sectors, where tensions and synergies emerge beyond the individual level. More awareness of how different organisational structures are involved in shaping cross-sector service provision is needed. Differing payment structures and their impact on cross-sector working should be explored further. The focus has largely been at the micro/provider level but the interface between policies related to the independent sectors has not been explored. How these policies shape cross-sector service provision is unknown. Future research could explore what it is like for the leaders to lead their own teams while needing to consider other sectors in how they provide service to consumers. In addition, what is known about the difference between cross-sector service provision for chronic, as opposed to acute and short-lived conditions, is missing from the literature. There is likely variation in how the acuity of the concern shapes service provision across sectors, but little to nothing is reported about this in the literature.

### Limitations

The focus of this umbrella review was on service provision between two or more interconnected, yet *independent* sectors. Integration has come to be understood as existing on a continuum ranging from loose coordination to more overlap with a shared governance structure [28]. All appropriate studies related to integration were included in this umbrella review; however, it is worth mentioning that it was difficult to discern the extent of integration of all articles included in the existing reviews and this may have skewed the result of the current umbrella review slightly. However, excluding integration studies based on this concern would have posed a bigger threat to the overall integrity of the review. Despite lacking certain information, we felt it was important to include studies that might have been deemed lower quality because they still provided useful information regarding cross-sector service provision. Given the year range of articles included in the existing systematic reviews, it is possible that studies were included in more than one review, which could potentially influence the findings of the current umbrella review. The current authors could not do a comparison of included articles because authors of the included reviews did not always provide this information. Finally, only studies conducted in English were included in this umbrella review; therefore, we likely missed important studies conducted in other languages. As an example, we could not include a German review by Schmid, Steinert, and Borbe [30].

## Conclusion

The literature shows that the focus is still at the individual provider level, more so than the sector level. Further investigation into what is involved in developing a shared vision of care across diverse sectors is needed. This will undoubtedly include the consumer, front-line staff and leadership of each sector but should take a higher level look at what organisational facilitators and challenges exist at the boundary between sectors in regard to cross-sector service provision. The findings from the vast amount of literature in the area has aligned with the following: taking a client-centred approach, developing a shared vision of care, enhancing and supporting communication across sectors involved with cross-sector service provision and navigating power differentials. The findings from this umbrella review provide much needed insight into the role that individuals involved with cross-sector service provision play in the success and failure of the arrangements. However, future research from a cross-organisational perspective is needed to better understand what shapes cross-sector service provision. Highlighting substantial similarities to the work done a decade prior by Sloper [10] is not meant to imply that no progress has been made in the years since that review was conducted, but it does raise some concerns related to duplicating approaches that have previously been explored at length. Future researchers should focus on novel aspects that advance our understanding of cross-sector service provision.

## Competing Interests

The authors declare that they have no competing interests.

## Footnotes

We use the term ‘sector’ to refer to divisions of the healthcare and social care industries that are distinct from one another with regard to structure.

The authors of the current article will use the ‘primary term’ when referring to the terms specified by the authors of the included review articles. They include: Integration, Collaboration, or Partnership and articles will be categorised this way for the remainder of the current article. It is important to note that often the authors not only adopted a primary term but also used numerous different terms interchangeably throughout the individual articles.
